# Supplementary figures and images for: A more accurate method for colocalisation analysis allowing for multiple causal variants
Source: PLoS Genet. 2021 Sep 29;17(9):e1009440. doi: 10.1371/journal.pgen.1009440 (PMC8504726; doi:10.1371/journal.pgen.1009440)

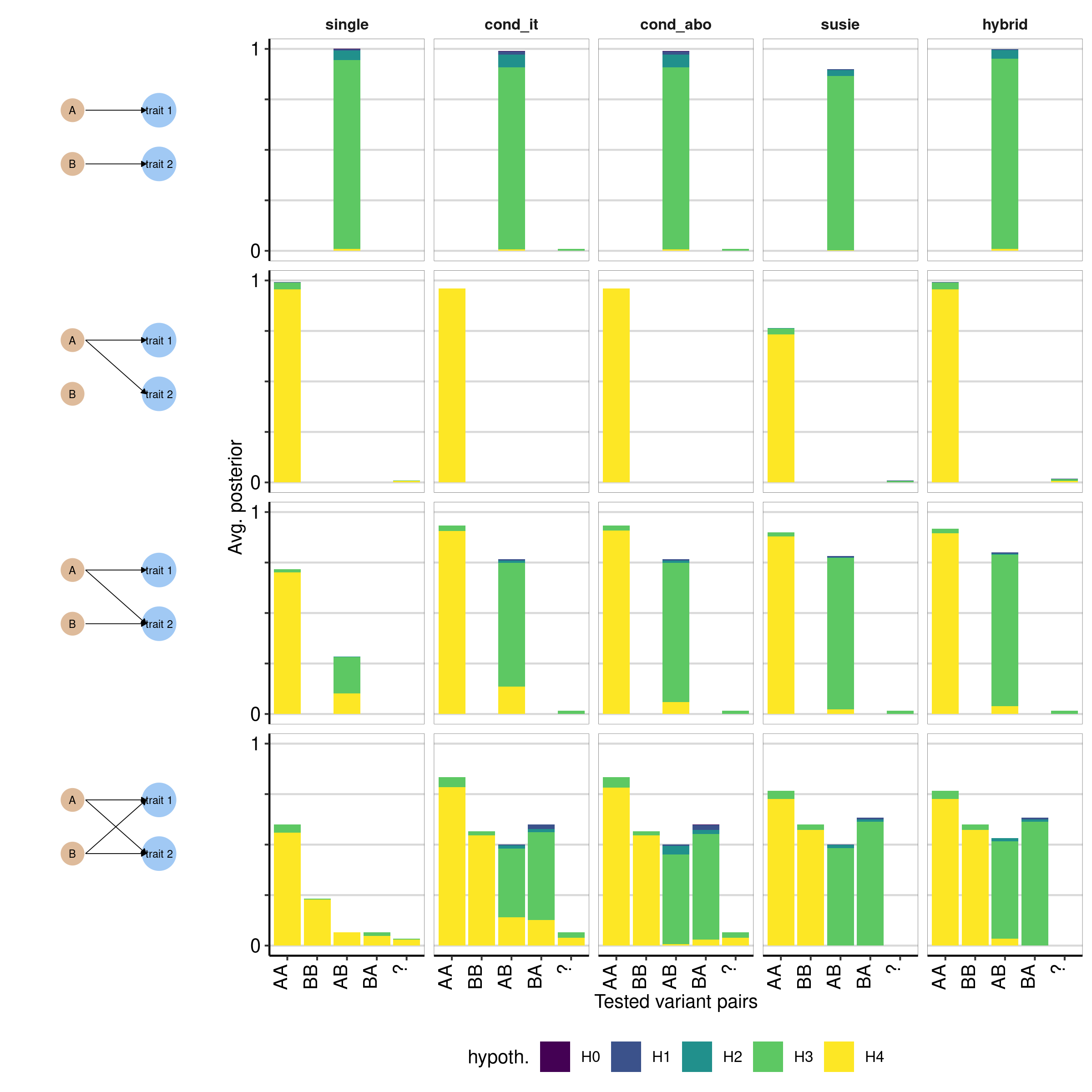

Supplement: S1 Fig — Legend otherwise as for Fig 1. (TIF) [file pgen.1009440.s002.tif]

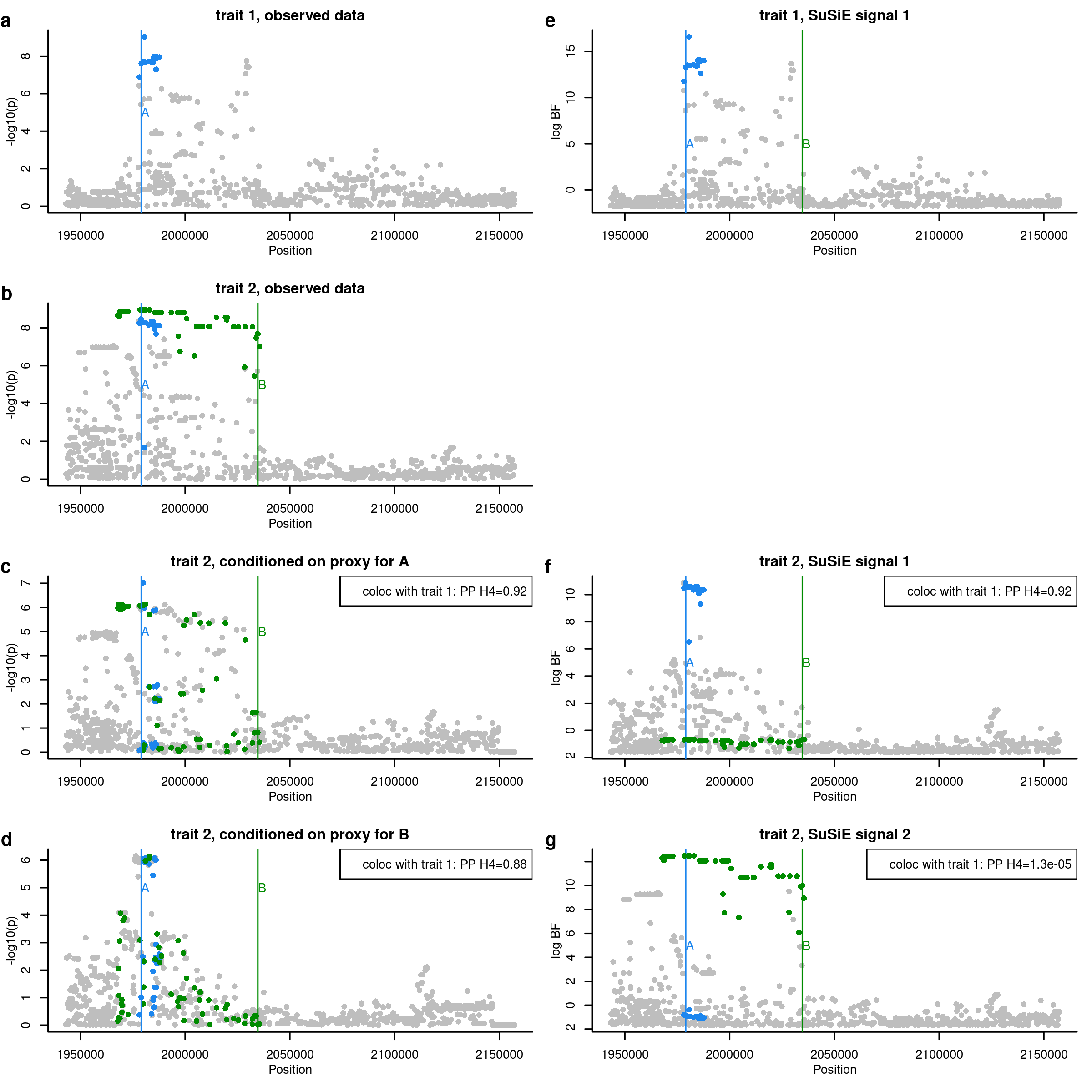

Supplement: S2 Fig — a and b show the observed data (-log10 p values) for traits 1 and 2 respectively. Conditioning identifies two independent signals for trait 2, and the results of conditioning on the signal closest to causal variants A and B are shown in c and d respectively. Coloc comparisons are based on (a, c) and then (a, d). SuSiE analysis of the same data finds one signal in trait 1, and log10 Bayes factors (BF) for this signal are shown in e. It finds two signals for trait 2, and the log10 BF for these are shown in f and g. Coloc comparisons are based on (e, f) and (e, g). The boxes on the lower plots show the results of running coloc analysis on that dataset against the data for trait 1 shown in a or e as appropriate. The data underlying this figure are available in S1 Data. (TIF) [file pgen.1009440.s003.tif]
