# Supplementary material for: A more accurate method for colocalisation analysis allowing for multiple causal variants
Source: PLoS Genet. 2021 Sep 29;17(9):e1009440. doi: 10.1371/journal.pgen.1009440 (PMC8504726; doi:10.1371/journal.pgen.1009440)
Supplement: Attachment — Submitted filename: coloc-susie response.pdf [file pgen.1009440.s006.pdf]

Reviewer #1:

This is a promising methodological contribution to the colocalization problem in GWAS. The central idea is intruiging, and the key results support the idea, but the way the results are presented is confusing, and the ideas and results raise a few questions.

Here are my main comments:

My colleagues (who helped me with parts of this review) and I had trouble following Fig. 3. Our confusion may be in part due to several typos. In the second row should there be an arrow from A to 2 rather than B to 1? In the third row should there be an arrow from A to 2 and no arrow from B to 1? And the sentence toward the end of Results should instead read, “susie seems to resolve this issue, with AB-like comparisons clearly having strongest posterior support for H3” (not H4)? Some explanations are missing, such as the bars in the plots (AA, BB, AB, etc), and how the key statistical quantities computed in coloc (the Bayes factors and posterior odds) relate to the plots (in particular, the heights of the different coloured bars). Also, the total height of the bars seems important, perhaps relating to power in some way, which is alluded to in the caption, but isn’t clearly explained.

> I apologise for the typographical errors in Fig 3. The arrows in the second and third rows were indeed switched. This has now been corrected, as shown below.

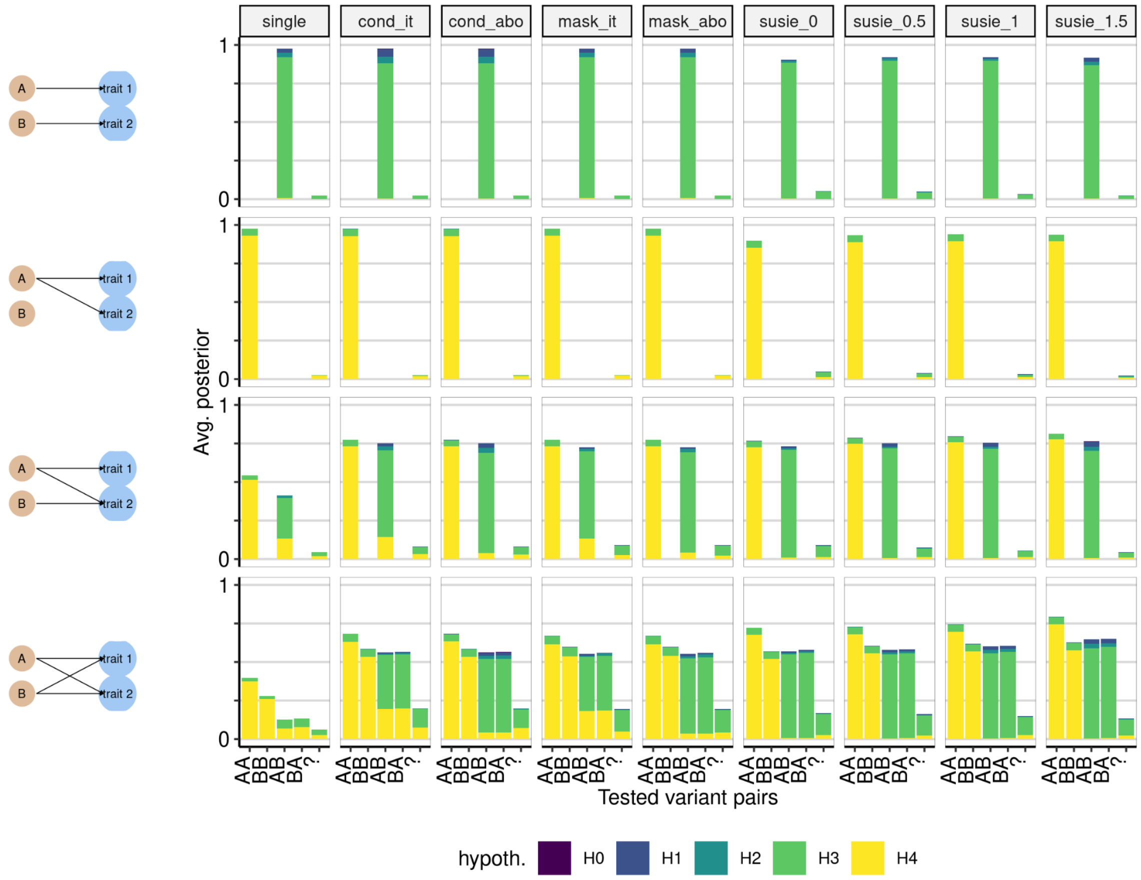

> The legend of the figure has been revised to better explain the heights of the different coloured bars and the total height, and also reduced to show only simulations of regions with 1000 SNPs, with the full set of simulations shown in Supp Fig 2 as suggested below.

Fig 3. Average posterior probability distributions in simulated data. The four classes of simulated datasets are shown in four rows, with the scenario indicated in the left hand column. For example, the top row shows a scenario where traits 1 and 2 have distinct causal variants A and B. Columns indicate the different analysis methods, with susie_x indicating that SuSiE was run with data trimmed at |Z|< x, cond_it indicating that conditioning was run in iterative mode, and cond_abo indicating it was run in "all but one" mode. For each simulation, the number of tests performed is at most 1 for ``single", or equal to the product of the number of signals detected for the other methods. For each test, we estimated which pair of variants were being tested according to the LD between the variant with highest fine-mapping posterior probability of causality for each trait and the true causal variants A and B. If r^2>0.5 between the fine-mapped variant and true causal variant A, and r^2 with A was higher than r^2 with B, we labeled the test variant A, and vice versa for B. Where at least one test variant could not be unambigously assigned, we labelled the pair ``?". The total height of each bar represents the proportion of comparisons that were run, out of the number of simulations run, and typically does not reach 1 because there is not always power to perform all possible tests. Note that because we do not limit the number of tests, the height of the bar has the potential to exceed 1, but did not do so in practice. The shaded proportion of each bar corresponds to the average posterior for the indicated hypothesis, defined as the ratio of the sum of posterior probabilities for that hypothesis to the number of simulations performed. Each simulated region contains 1000 SNPs.

If we are interpreting the bars correctly, I was confused by the AB / BA signals. I think the key is when you assume one causal per region then AB / BA will inevitably capture some H4 because AB / BA are simply wrong models. In short, some of the bars may be more important than others to "get right", and the way it is presented it may be hard for the reader (and reviewer!) to focus on the differences across methods that are important—by "important," differences that would matter in practice when applying coloc to real data sets. For example, in the 4th scenario (4th row of Fig. 3), the results for "cond", "mask" and "susie_0" are pretty much the same, except for the case of AB and BA. The question is whether these differences among the methods matter in this case? The level of detail in Fig. 3 may be useful for getting a better understand for the behaviour of the different approaches, but is there a way to summarize these results in an evocative way, say, using ROC or precision-recall curves?

The legend now also, hopefully, better explains the process of arriving at AB and BA signals. For each simulation, method "single" performs at most one test. The number of tests for all other methods is determined by the number of signals they each detected for each trait, and all pairs of detected signals are compared. A test is assigned to the "AB" category if the SNP with the highest posterior probability of causality for trait 1 has r2>0.5 with the true "A" causal variant, and the SNP with the highest posterior probability of causality for trait 2 has r2>0.5 with the true "B" causal variant.

The issue in the 4th row with apparent AB tests giving some posterior support to H4 for cond and mask relates to a "feature" of coloc which has been considered helpful before under a single causal variant assumption. If trait 1 has two causal variants A and B, and trait 2 has only B, then you still expect high posterior support for H4. You only expect a high posterior for H3 when the two traits share 0 causal variants. I think this is part of why coloc has continued to be used despite the single causal variant, because it will detect sharing if it exists, even in the presence of additional non-shared effects.

However, in the conditional case, this “feature” can itself cause issues, which is a point I made too subtly before. I have now tried to explain by walking through a couple of examples in the Results

This feature also presents problems for the conditioning approach, as demonstrated by the high average posterior probability for H_4 in the ``AB'' comparisons, one of which is examined in detail in Fig 4. In this example, trait 1 has one causal variant, A, whilst trait 2 has two, A and B, with B having slightly greater significance. In the first round of analysis by the conditioning method, the original sets of summary statistics are passed to coloc. Because A is the stronger effect for trait 1, the test is labelled ``AB'', but gives a high posterior to H_4 because there is one shared causal variant (A). Then the stronger effect, B, is conditioned out, and the analysis rerun with trait 1, and trait 2 conditioned on B. This test again gives a high posterior for H_4. This situation is confusing, because the same signal in trait 1 appears to colocalise with different signals in trait 1. SuSiE models both signals simultaneously, so we can attempt to colocalise trait 1 with each signal independently, finding high H_3 for one and high H_4 for the other. If we were confident we could infer both the exact number of independent signals and their identity correctly by conditioning, we could attempt to emulate this in the conditioning, using the ``all but one'' rather than ``iterative'' mode. This does result in better average performance than the iterative mode (Fig \ref{fig:simstrat}). However it is often outperformed by SuSiE. Supplementary Fig 3 shows an example where the stepwise approach is less able to correctly identify the separate signals. The A signal is not well identified, and therefore not be adequately conditioned out, which may results in two apparently different comparisons with trait 1 which both produce a high H_4. In this example too, SuSiE more correctly produces two comparisons, one with high H_3 and one with high H_4.

Figure 4. Example where the conditional coloc approach, run in iterative mode, finds misleading results. \textbf{a} and \textbf{b} show the observed data (-log p values) for traits 1 and 2 respectively. Conditioning identifies a second independent signal for trait 2, and the results of conditioning on the strongest signal is shown in \textbf{c}. Coloc comparisons are based on (a,b) and (a,c) and both find the posterior probability (PP) of H_4 is >0.8. SuSiE analysis of the same data finds one signal in trait 1, and log Bayes factors (BF) for this signal are shown in \textbf{d}. It finds two signals for trait 2, and the log BF for these are shown in \textbf{e} and \textbf{f}. Coloc comparisons are based on (d,e) and (d,f) and find PP of H_{4} of >0.9 and <10^{-4} respectively. Blue and green points are used to highlight SNPs in LD with (r^2>0.8) the true causal variants A and B respectively.

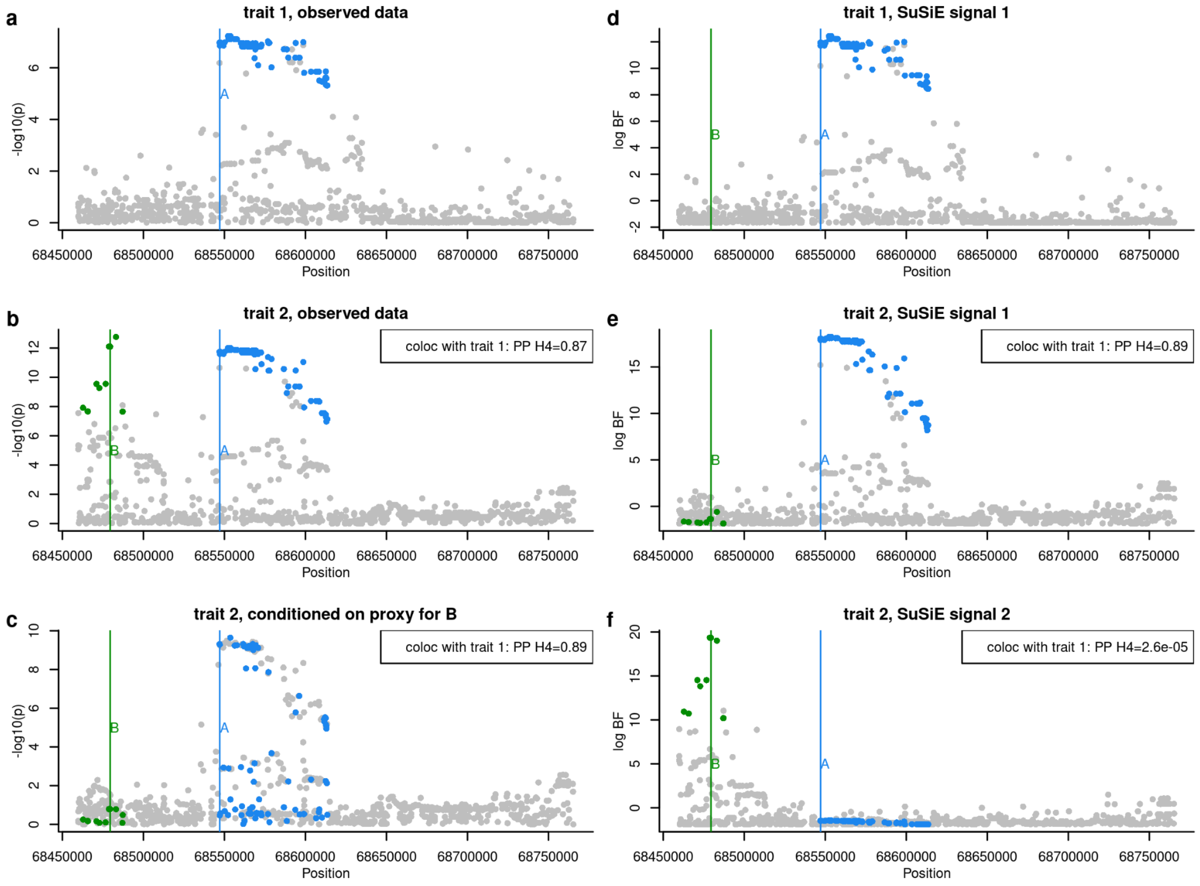

A minor point is the results do not seem to differ much across different numbers of SNPs (1000, 2000, 3000) and I wonder if it would be better to show the results for only one setting, and move the other two settings to the supplement).

> Agreed, this is now done.

There is an early emphasis on addressing the issue that susie is too slow to be run on large regions. On the surface, it seems reasonable to "trim" SNPs based on some safe cutoff (e.g., based on p-values). However, some elaboration is needed to justify having to take this step; consider that the computational complexity of susie is linear in the number of samples (n), SNPs (p), and number of "single-effects" (L), so in principle susie should be able to handle large data sets, with available memory typically being the greatest limitation. (We have run susie on data sets with as many as 12,000 SNPs.) Also, for data sets with large n, there is a summary-statistics version of susie (susie_rss). I'm not arguing against the trimming per se, but to motivate these steps it would be helpful to explain in more detail the challenges faced in using susie, for example, whether the high computational expense was due to slow convergence (which could happen when SNPs are very strongly correlated), or due to something else. I wonder if this trimming is in fact avoidable.

> This was motivated by my group's attempts to run this version of coloc (which uses susie_rss) across multiple pairs of datasets genomewide. We found some regions with large numbers of snps (p) could take many hours to run, so wanted to investigate whether there was a workaround to speed this up. Reviewer 2 notes that the eigen decomposition required for the summary statistics function which we use,

susie_rss, is O(p^3) where p is the number of SNPs, and perhaps if you are running on full genotype data this explains our different experiences? I now have expanded the discussion to include more information on the computational complexity of susie_rss, the alternative solutions which could be explored, and an explicit warning that results about trimming with coloc do not transfer to the fine-mapping setting.

Despite the adoption of a novel iterative procedure to fit the SuSiE model, the procedure is still slow for large regions with many SNPs, which can be a barrier to its adoption for a technique like coloc which has always boasted speed as an advantage. We note that the package susieR is still being developed. The most computationally expensive step in susie_rss is the eigen decomposition of the LD matrix, which is O(p^3) where p is the number of SNPs. In the case where multiple pairs of traits are being considered for colocalisation, computing this decomposition once in advance could be used instead to improve speed. Alternatively, it may be that with standardised datasets covering the same sets of SNPs, such eigen decompositions could be precomputed and stored. Finally, further development of susie_rss may lead to avoiding the eigen decomposition step altogether.

In this manuscript, we considered a simple approach, approximating the SuSiE posterior by using a trimmed set of data, discarding SNPs with |Z| scores below some small threshold, on the assumption that a causal SNP with detectable association should produce a Z score of reasonable magnitude. (For reference, whilst we only consider discarding SNPs with |Z|<1.5 at most, the standard genome-wide significance threshold of p<5\times10{-8} corresponds to |Z|>5.45). Thus, this approximation makes the assumption that true causal variants will have at least some weak marginal evidence of association. We note that it is possible to construct examples which will violate this assumption, for example if two causal variants in strong LD but with opposite directions of effects exist. Further, in simulated data here, we found that trimming can increase false positive signals when fine-mapping, a phenomenon previously noted (Sesia et al., 2020). Thus we suggest that trimming is inappropriate when fine-mapping is the end-goal of any study, especially given that false positives in fine-mapping may result in substantial costs if followed-up in wet-lab experiments. However, these fine-mapping false positives did not appear to increase false positives in coloc, perhaps because the occurence of false positives was relatively rare, such that it did not occur often in both members of a pair of datasets, and/or perhaps because when false positives did occur, they were focused on different SNPs in the two datasets so were unlikely to generate support for H_4, the hypothesis of most interest. Given this, we suggest a threshold of |Z|<1 may be acceptable to allow SuSiE coloc to run at speed in larger regions, but leave the threshold as a user-set parameter which is 0 by default, and which we recommend should be reported along with any results. For the most noteworthy results, we recommend analysis should be repeated without trimming to ensure inference is robust to the approximation.

A larger question is raised by relaxing the assumption of one causal variant, and it surfaces as one attempts to define the colocalization problem. Perhaps it can be addressed by more careful definitions. For example, you state in the introduction, “Colocalisation is a technique used for assessing whether two traits share a causal variant in a region of the genome, typically limited by LD.” But when allowing for the possibility of multiple causal variables, hypotheses H0–H4 no longer cover all possible events. For example, perhaps H4 should be defined as, *“both traits are associated and share at least one causal variant?”* In short, based on the description you have given, there appears to be a mismatch between the coloc inferences and the susie inferences, which is to compute a posterior distribution over all 2^p combinations of causal variables (with the constrained that at most L SNPs can be included).

That is—if I am not misunderstanding—coloc makes inferences at the SNP level, and susie makes inferences at the region level. I don't see this necessarily as a fundamental issue, but more an issue of being more precise in definitions (and perhaps reminding us—the readers and reviewers—what coloc is actually doing).

> Thank you - this is a very good point. I have expanded in the Introduction on the earlier re-framing of the colocalisation problem to the multiple causal variant situation, by assuming that data can be decomposed into layers corresponding to the distinct causal variants. The mechanism for doing so, stepwise regression, has established weaknesses. The central goal of the current paper is to assess whether the new SuSiE approach to this decomposition is beneficial to colocalisation.
>
> *This simple summation is enabled by the single causal variant assumption, which implies that each pair of variants being causal for the two traits are mutually exclusive events. However, the assumption is unrealistic, as multiple causal variants may exist in proximity, which also challenges the definition of colocalisation as presented above as none of the global hypotheses encompass multiple causal variants.*
>
> *In previous work, (Wallace 2020) we allowed for multiple colocalisation comparisons to be performed in a region, each labelled by a pair of SNPs tagging each of the distinct causal variants for each trait. Thus, if trait 1 had two causal variants tagged by SNPs A and B and trait 2 had one, tagged by SNP C, we would conduct two colocalisation analyses, to ask whether A and C corresponded to a shared causal variant, and whether B and C corresponded to a shared causal variant. This allows the simple combination of Bayes factors through summation, but explicitly assumes that data can be decomposed into layers corresponding to the causally distinct signals. The stepwise regression approach upon which conditioning is based is known generally to produce potentially unreliable results (Miller 1984), a phenomenon that can be exacerbated by the extensive correlation between genetic variants caused by linkage disequilibrium (LD) (Asimit et al 2019).*
>
> *A suite of Bayesian fine-mapping methods have been developed recently which calculate posterior probabilities of sets of causal variants for a given trait (Benner et al 2016, Newcombe et al 2016, Hormozdiari et al 2014). However, the marginal posterior probabilities calculated from these are no longer mutually exclusive events, so they could not be easily adapted to the colocalisation framework. An alternative would be to consider all possible combinations of models between two traits, but this combinatorial problem is computationally expensive (Asimit et al 2019). Recently, the Sum of Single Effects (SuSiE) regression framework (Wang et al 2020) was developed which reformulates the multivariate regression and variable selection problem as the sum of individual regressions each representing one causal variant of unknown identity. This allows the distinct signals in a region to be estimated simultaneously, and enables quantification of the strength of evidence for each variant being responsible for that signal. Conditional on the regression being considered, the variant-level hypotheses are again mutually exclusive. Here we describe the adaptation of coloc, allowing for multiple labelled comparisons in a region, to use the SuSiE framework and demonstrate improved efficacy over the previously proposed approaches.*

Finally, the simulation setup lacks detail, e.g., Which 1kg data set was used? How many 1000 Genomes samples were used, and from which geographic regions? How were the effects and traits simulated? How were the regions chosen (e.g., are they regions near gene coding regions)? What are the MAFs of the SNPs?

I have added the following to the “Simulation strategy” section in the Methods

We examined the performance of the approximation described above to decrease the computational burden, and of using SuSiE for colocalisation by simulation.

We used lddetect (Berisa et al, 2016) to divide the genome into approximately LD-independent blocks, and extracted haplotypes from the EUR samples in 1000 Genomes phase 3 data, consisting of 1000 contiguous SNPs with MAF > 0.01. We simulated case-control GWAS summary statistics for a study with 10,000 cases and 10,000 controls, corresponding to the LD and MAF calculated from these haplotypes using simGWAS (Fortune et al, 2018), with one or two common causal variants (MAF > 0.05) chosen at random and log odds ratios sampled from N(0,0.2^2). We discarded any datasets which did not have a minimum p<10^{-6} to match our expectation that fine-mapping and colocalisation are only conducted when there is at least a nominal signal of association. We simulated 100 such datasets for each of 100 randomly selected LD blocks, and sampled from these sets of summary data for all the simulations detailed below.

Reviewer #2:

Summary

This paper introduces an extension of the "coloc" method for colocalization to deal with multiple causal variants in a region. This extension exploits a recently-introduced method for fine mapping (SuSiE). The extension is attractive in its simplicity, and simulations show it to perform better than some alternative approaches. The paper also suggests a way to speed up computations by pre-filtering out "non-significant" SNPs.

The key idea of combining SuSiE and coloc is nice, and I think that with some improvements to the presentation will make a nice publishable contribution.

The idea of speeding up SuSiE by pre-filtering SNPs is also attractive from a practical point of view, but it has some potential downsides that I feel are not sufficiently emphasized and explored (even though the manuscript does end with a statement that trimming might be not beneficial in general final mapping). Specifically trimming out non-significant SNPs could increase the potential for false positive identifications, and indeed such a result has been previously reported in https://www.biorxiv.org/content/10.1101/631390v3 (their Figure S7). It's not clear to me how, if at all, this is reflected in the results shown here. Maybe it is simply the case that, as the paper suggests in the discussion, that "Coloc benefits from comparing posterior probabilities across... two traits".

But the overall way that the manuscript deals with false positive (or indeed false negative) identifications is not clear. (Maybe methods are applied with some knowledge of the true number of causal effects? It isn't clear to me.)

Since there are also other potential ways to speed up computation (see comments below) I am not really convinced that the pre-filtering approach is really the way to go, and would like to see at least a stronger assessment of the potential downsides.

Thank you for the detailed review. I think all the points here are expanded on below, so please see the responses to your specific points there.

Main Comments

1. The presentation of the method requires more details, including more precise equations showing how quantities computed by SuSiE are used/combined. For example you could introduce α_{lj} for the matrix of posterior probabilities output by susie and then give explicit expressions for the Bayes Factors being computed (BF_{lj}) in terms of α_{lj}. I'm not sure what P_0 is (is it something output by SuSiE?)

Is $\pi=1/p$ where p is the number of SNPs in the region, or something else? How do you set the maximum number of effects in SuSiE (L in the SuSiE paper)? Do you get SuSiE to estimate the number of effects by estimating the prior variance, or do fix the prior variance?

If L_g is the number of effects identified by SuSiE in the GWAS and L_e the number identified by SuSiE in the eQTL study, do you end up running coloc $L_g * L_e$ times? (as suggested by "for every pair of regressions across traits" on p3).

How do you combine/summarise the results from all these different runs of coloc?

I have adjusted coloc to use the Bayes factors that are now returned by susie_rss (thank you), so the back-calculation is no longer needed and I have removed that equation. I have also updated the relevant text to give more details on how susie_rss is called, and the number of colocalisation comparisons returned and how the pairwise probabilities of colocalisation may be interpreted:

The new coloc.susie function in the coloc package (https://github.com/chr1swallace/coloc/tree/susie) takes a pair of summary datasets in the form expected by other coloc functions, runs SuSiE on each and performs colocalisation as described below. We use the susie_rss() function in the susieR package to fine-map each summary statistic dataset, run with default options, although the \texttt{susie.args} argument in coloc.susie() allows arguments to be supplied to susie_rss(). SuSiE returns a matrix of variant-level Bayes factors for each modelled signal and a list of signals for which a 95% credible set could be formed, corresponding to a subset of rows in the matrix of Bayes factors. These rows are then analysed in the standard coloc approach, for every pair of regressions with a detectable signal across traits. Explicitly, if L_1 and L_2 signals are detected (have a credible set returned) for traits 1 and 2 respectively, then the colocalisation algorithm is run $L_1 \times L_2$ times. Thus, the user is presented with a list of tag SNPs per signal for each trait, and the matrix of pairwise posterior probabilities of H_4 may be examined to infer which, if any, pairs of tags represent the same signal.

2. Presentation of colocalization results also needs more details. Can you say explicitly what is an "AA" or "BB" comparison and an "AB-like signal"? From the description on p3 I thought the simulations would include settings where there were 2 causal variants in each trait, but no sharing. But Fig 3 seems to suggest only a small portion of potential configurations of up to 2 signals in each trait are actually included - is that right? (why?) And in Fig 3, what happens if SuSiE finds a signal in one trait and not in the other - what comparison do you make? (Or do you force SuSiE to find the right number of effects in each trait by fixing L to the true value? If so, is that cheating?) Is the smaller height of the AA bar for susie_0 compared with other methods -- and indeed the slightly smaller height of all bars -- something to be concerned about? Are all methods equally applicable if (as is always the case) you do not know the true number of causal signals in each trait?

> I completely agree it would be cheating to tell any method how many causal variants were simulated! I have updated the legend to figure 3, which hopefully clarifies what AA, BB etc signals are.
>
> *Fig 3. Average posterior probability distributions in simulated data. The four classes of simulated datasets are shown in four rows, with the scenario indicated in the left hand column. For example, the top row shows a scenario where traits 1 and 2 have distinct causal variants A and B. Columns indicate the different analysis methods, with susie_x indicating that SuSiE was run with data trimmed at |Z|< x, cond_it indicating that conditioning was run in iterative mode, and cond_abo indicating it was run in "all but one" mode. For each simulation, the number of tests performed is at most 1 for ``single'', or equal to the product of the number of signals detected for the other methods. For each test, we estimated which pair of variants were being tested according to the LD between the variant with highest fine-mapping posterior probability of causality for each trait and the true causal variants A and B. If r^2>0.5 between the fine-mapped variant and true causal variant A, and r^2 with A was higher than r^2 with B, we labeled the test variant A, and vice versa for B. Where at least one test variant could not be unambigously assigned, we labelled the pair ``?''. The total height of each bar represents the proportion of comparisons that were run, out of the number of simulations run, and typically does not reach 1 because there is not always power to perform all possible tests. Note that because we do not limit the number of tests, the height of the bar has the potential to exceed 1, but did not do so in practice. The shaded proportion of each bar corresponds to the average posterior for the indicated hypothesis, defined as the ratio of the sum of posterior probabilities for that hypothesis to the number of simulations performed. Each simulated region contains 1000 SNPs.*
>
> In the setting where each trait has 2 causal variants A and B, there should ideally be 4 comparisons: AA, AB, BA, BB. Very occasionally there are more (a handful of times susie_rss reports > 2 signals). Often there are fewer (due to power), and even when there are 4, they may not all be assignable to labelled groups and some will fall in "?". This is why the bar height, which is proportional to the number of comparisons performed in a given category divided by the number of simulations, doesn't reach 1.

3. Figure 1 compares only the PIPs at causal variants. Since in practice we don't know the causal variants, one should also care about PIPs at non-causal variants. Is there a tendency for SuSiE to inflate PIPs at non-causal variants when trimming?

> Figure 1 has now been changed to show both the change in PIP at the causal variants and at the non-causal variants. Specifically, following the approach of coloc

to consider comparisons between vectors of Bayes factors corresponding to detected credible sets, I took the colSums of obj$alpha[obj$sets$cs_index,] as a measure of the total PIP after a run of susie_rss(). Thus the total PIP can increase/decrease by 1 if a credible set is “discovered” or “lost” after trimming. This view of the data shows false positives are indeed introduced after trimming. This is now described at the beginning of the Results section

First we assessed the impact of trimming data on the accuracy and speed of SuSiE. We found that trimming had a very small effect on PIP estimates at the causal variants (Fig 1). Interestingly, when estimates did change, they were more likely to detect a true signal after trimming than lose a true signal (approximately 1-2% of simulations related led to true causal variants that were discovered only after trimming, while <= 1% of simulations led to true causal variants being discovered in the full data but not in the trimmed data). False signals were also more likely to be detected after trimming, however. This was more extreme with larger |Z| thresholds and in simulations with two rather than one causal variants, when over 2% of simulations resulted in signals being detected at non-causal variants after trimming at |Z|<1.5. One might expect the situation to only worsen as the number of causal variants increases. Thus, trimming is expected to introduce false positives at a higher rate than it might increase detection of true positives, although it did reduce the median time for a SuSiE run per region more than ten fold (Fig 2).

Figure 1. Difference in the sum of estimated PIP at the causal variant(s) (y-axis) versus non-causal variants (x-axis) between analysis with the full model and data trimmed to |Z| above some threshold. The percentage of 2000 simulations falling in each region is shown to the nearest 1 decimal place (note that ``0'' indicates <0.05%). Marginal densities show the concentration of observations in either direction around (0,0). Datasets all had 1000 SNPs, but differ in the number of causal variants (1 or 2) and the |Z| threshold used for trimming.

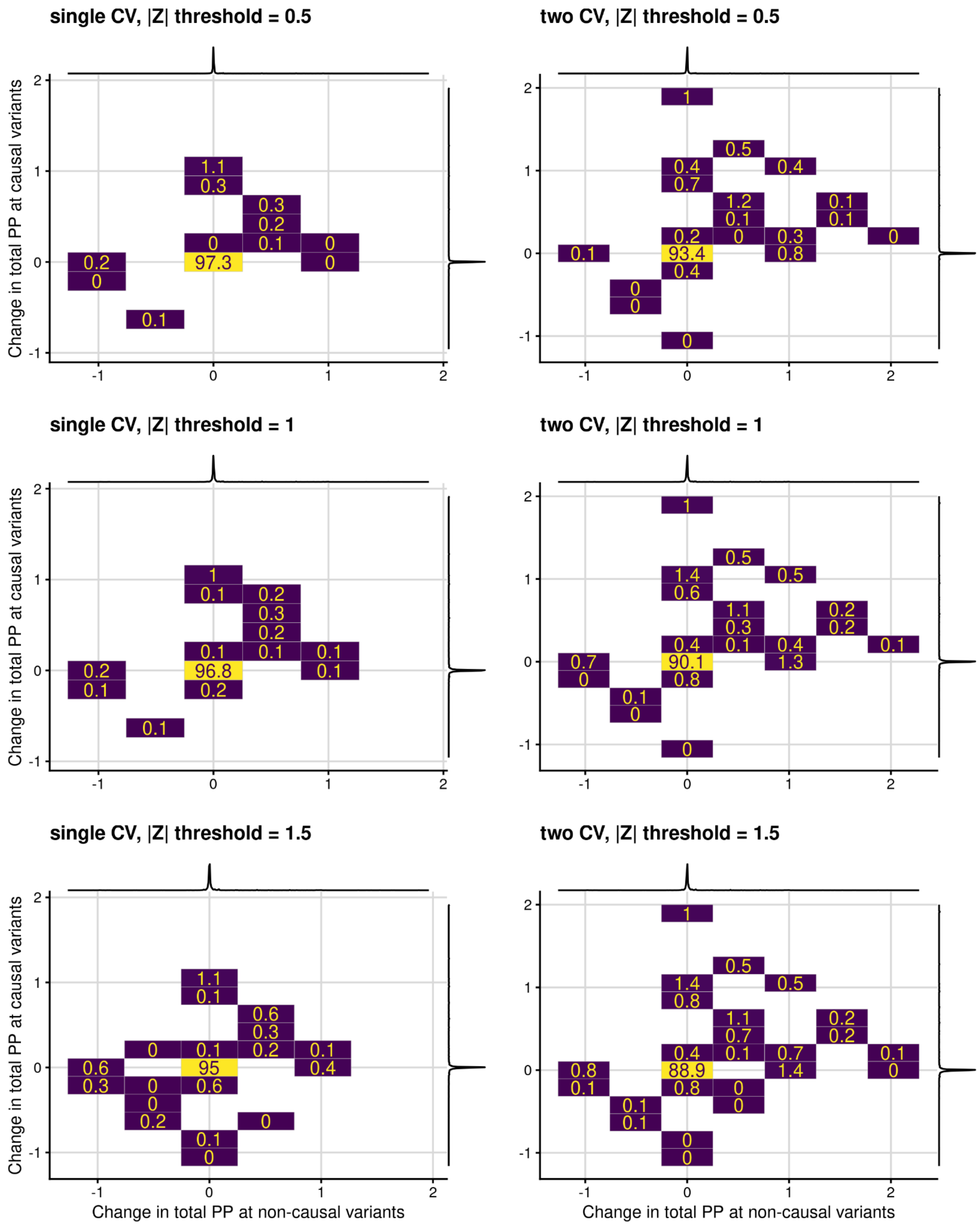

Fig 3 shows, however, that despite these false positives coloc with SuSiE outperforms the alternatives, and appears to do at least as well when trimming as not. Therefore, I expanded the Discussion on trimming to give a clearer warning that while trimming is possible for coloc, these results do not support its use in fine-mapping.

...in simulated data here, we found that trimming can increase false positive signals, a phenomenon previously noted (Sesia et al, 2020). Thus we do not suggest that

> *trimming is appropriate when fine-mapping is the end-goal of any study, especially given that false positives in fine mapping may result in substantial costs if followed-up in wet-lab experiments. However, these fine-mapping false positives did not appear to increase false positives in coloc, perhaps because the occurence of false positives was relatively rare, such that it did not occur often in both members of a pair of datasets, and/or perhaps because when false positives did occur, they were focused on different SNPs in the two datasets so were unlikely to generate support for H_4, the hypothesis of most interest. Given this, we suggest a threshold of |Z|<1 may be acceptable to allow SuSiE coloc to run at speed in larger regions, but leave the threshold as a user-set parameter which is 0 by default, and which we recommend should be reported along with any results. For the most noteworthy results, we recommend analysis should be repeated without trimming to ensure inference is robust to the approximation.*

4. It seems there are many potential ways to improve computation than filtering out non-significant SNPs, and many of them may ultimately be better choices (although filtering is obviously very simple to implement!) I don't think the discussion in the paper really adequately reflects the options available or the many issues involved.

Although I did not see it explicitly said anywhere, I believe the paper is using the susie_rss function for applying SuSiE to summary data. The details of this function are not included in the original SuSiE publication, but at time of writing this function works by performing an initial eigendecomposition of the reference LD matrix R, which makes it possible to convert the summary data into "transformed data" to which regular SuSiE can be applied. This approach is appealing from a software engineering point of view, but not necessarily the most efficient, computationally. The eigendecomposition of R is quite expensive, being O(p^3) where p is the number of SNPs.

The subsequent application of SuSiE to the transformed data is O(p^2) per iteration.

Thus if p is sufficiently large the eigendecomposition step will likely dominate the susie_rss computation (and Figure 2 does indeed suggest computation maybe increase something like p^3?)

One way to reduce computational complexity would therefore be to avoid the eigendecomposition step, and we are currently actively exploring these in our development of susie_rss.

However, note that computing R itself is already an O(np^2) operation, where n is the number of samples in the reference sample used to compute R. So if n is big then this computation (which is basically considered free in this paper since R is precomputed) could be the dominant computational cost. Alternatively

if n<in the case n<

SVD of the reference genotypes (O(n^2p)) which will cheaper than forming R (O(np^2)) when n<In the future it seems quite likely that pre-computed R and eigen(R) could be made

available for some large panels, avoiding the need for each user to compute them. Once these pre-computations are done there may no longer be any need to filter SNPs.

Thank you for these detailed explanations. I agree it would be preferable to never trim data. I have extended the discussion to cover these points, and acknowledged your comments in helping me to do that.

We note that the package susieR is still being developed. The most computationally expensive step in susie_rss is the eigen decomposition of the LD matrix, which is $O(p^3)$ where p is the number of SNPs. In the case where multiple pairs of traits are being considered for colocalisation, computing this decomposition once in advance could be used instead to improve speed. Alternatively, it may be that with standardised datasets covering the same sets of SNPs, such eigen decompositions could be precomputed and stored. Finally, further development of susie_rss may lead to avoiding the eigen decomposition step altogether.

Other comments/details

- p3 although the number of potential models increases exponentially, SuSiE computation does not increase exponentially.

Corrected by removing the link between the increased computation with p and the number of models.

- p4: "We labelled each comparisons considered...." I did not understand this sentence.

I have expanded this section:

In order to assess the accuracy of each coloc analysis, we needed to assess whether the comparison corresponded to a case of shared or distinct causal variants.

For each signal passed to coloc, we identified the variant with the highest posterior probability of causality, v_1 and v_2 for traits 1 and 2 respectively (it is possible that $v_1=v_2$). We then labelled the variant v_i ($i=1,2$) according to the rules:

A: $r^2(v_i,A) > 0.5 \land r^2(v_i,A) > r^2(v_i,B)$

B: $r^2(v_i,B) > 0.5 \land r^2(v_i,B) > r^2(v_i,A)$

-: otherwise

If either of the variants was labelled ``-" then the comparison was labelled ``unknown". Otherwise it was labelled by the concatenation of the two labels. We compared the average posterior probability profiles between methods, stratified according to this labelling scheme.

- p4: "... having strongest posterior support for H_4" - this should be H_3?

Yes, corrected.

- p8: " this does apply to single trait" - missing *not*?

Yes, though this sentence has now been rephrased.

- In the second row-set of Figure 3, is the figure on the LHS wrong? (The methods suggest colocalization but the figure shows no shared variant...)

Yes, corrected

- on p7 the r2 threshold is 0.8 but on p4 it is 0.5. Are there referring to different thresholds?

The 0.8 is a typo, now corrected.

Reviewer #3:

This is an interesting paper. The method is solid and implements M Stephen group's SUSIE method in the coloc framework with some simulation based comparisons with other methods (and "trimming" rather than shrinkage to help compute time). Expanding coloc to multiple variants is a useful advance to the field, and that is what PLoS Genetics Methods section papers are supposed to do.

I only have minor comments.

The formatting of figure 3 - the scenarios - seems to have gone slightly awry and needs to be fixed.

Thank you, now fixed

I suggest the discussion could be extended slightly - it is rather brief (although sufficient).

Now extended, partly to describe the choices made in extending coloc to multiple causal variants, in comparison to choices made by other approaches.

This manuscript presents one approach to colocalisation in the case of multiple causal variants, that assumes that distinct signals can be decomposed even if physically proximal, which SuSiE appears to do admirably well. This framing of the colocalisation problem implicitly assumes there are a finite number of causal variants for any trait which can be identified, and that traits may be compared in terms of their causal variants to identify shared variants. However, the concept of regional colocalisation can be approached in other ways in the multiple causal variant scenario. One approach reduces the possible hypotheses to two, with the alternative hypothesis corresponding to the existence of a causal variant in a region shared by two (or more) traits. (Deng et al 2020) Another focuses on a variant-level definition of colocalisation, estimating the probability that each variant in turn is causal for two traits, whilst allowing that other causal variants (shared or non-shared) may exist in the vicinity (Hormozdiari et al, 2016). In contrast, the approach proposed here allows the number hypotheses tested to be determined by the data. Whilst it relaxes the assumption of a single causal variant, one obvious caveat is that we have not yet reached (nor may we ever reach) sample sizes which enable all causal variants to be identified. Missed causal variants will provide incomplete comparisons of traits. It is also established that in lower power situations, even Bayesian fine-mapping methods

that simultaneously model causal variants may identify a single SNP which tags two or more causal variants (Asimit 2019) and the interpretation of non-colocalisation at such false signals is likely to be misleading. On the other hand, it does seem useful to go beyond asking whether at least one causal variant is shared, and the attempt to both isolate and count the distinct causal variants per trait may be useful in designing follow-up experiments. As we better understand the architecture of complex traits, and design methods that accomodate the multiple causal variants that have been discovered, it is important to bear in mind that results will continue to be limited by sample size, and limited ability to detect rarer variants or those in regions of particular allelic heterogeneity, which even sophisticated methods such as SuSiE may find challenging.
