# Supplementary material for: A more accurate method for colocalisation analysis allowing for multiple causal variants
Source: PLoS Genet. 2021 Sep 29;17(9):e1009440. doi: 10.1371/journal.pgen.1009440 (PMC8504726; doi:10.1371/journal.pgen.1009440)
Supplement: Attachment — Submitted filename: coloc-susie revised, response(1).pdf [file pgen.1009440.s008.pdf]

A note to both reviewers:

I thank you for taking another look at this manuscript. Whilst revising, I updated to the latest version of susieR on CRAN (0.11.42) and reran all analyses. I found results were produced considerably faster (average times to analyse a 3000 SNP region reduced from 80 seconds to 4 seconds). It is possible this relates to some configuration change on our HPC, but I suspect it is more likely that it relates to the newer version of susieR. I have therefore removed mention of the data-trimming approximation previously included, because there does not seem to be a need to improve on speed anymore. I have mentioned this change explicitly in the Discussion, because the preprint has already received some attention on Twitter, including of this "speed up approximation", and I wanted to be clear about why that had disappeared from this latest version:

"Note that in earlier preprints of this manuscript, we suggested an approach based on trimming input data to decrease the computational time required to run \texttt{susie_rss}, but more recent versions of susieR, including the one used here, are faster and so we no longer consider that approach to be required."

This has required a renumbering of figures (3->1, 4->3) and there are new figures 2 and 4 in response to comments by reviewer 1.

Comments to the Authors:

Reviewer #1:

I apologize for the delay in sending my feedback. The revised manuscript is improved and makes several points more clear, although there remain some gaps that need to be addressed. Please see below for my detailed comments. My intent is for my comments to be constructive and I hope they inspire further improvements.

> Thank you, the comments are indeed constructive.

Parts of the introduction remain imprecise. For example, you say, "This simple summation is enabled by the single causal variant assumption, which implies that each pair of variants being causal for the two traits are mutually exclusive events." I'm not sure what you mean by this? I think the main message is that the calculations of the BFs are much simpler under the single-variant assumption, which I agree with. Also, "The approach proceeds by enumerating all variant-level hypotheses — the possible pairs of causal variants (or none) for the two traits — and the relative support for each in terms of Bayes factors." But don't you allow (in susie) for more than 2 causal variants? Maybe introducing some simple mathematical expressions to define what is meant by the "single-variant assumption" and how the BFs are computed with and without the single-variant assumption would be helpful to make this more precise.

> I have revised the first two paragraphs of the Introduction again, hoping to clarify further and emphasising that all of this description relates to the single variant assumption form of coloc.
>
> I have not tried to explain the methodological detail however, because my previous attempts to introduce a mathematical description of coloc have always required a full exposition to make sense. The most recent exposition was published only last year. I have referenced this explicitly, which seems to me a better option than repeat it here.

Computational effort of fine-mapping with summary statistics. Quite a bit of the discussion in this manuscript is centered on susie being slow for large numbers of SNPs, and how to alleviate this issue. While not wrong, this discussion may be misleading. This issue is specific to using susie with summary statistics; one of the inputs to susie_rss will be a p x p matrix, where p is the number of SNPs. But, fundamentally, any regression-based fine-mapping method that takes as input a p x p matrix will have complexity at least O(p^2), making this an issue for any method that uses summary

statistics (of course, as you point out, because susie_rss computes an eigenvalue decomposition of the p x p matrix, the complexity is potentially greater than O(p^2), and there may be room to improve this). By contrast, with individual-level data susie's complexity is linear in n and p, which will be better than the summary data case when n < p. Related to this point, in the introduction please clarify that you are focussing on fine-mapping using summary data, and motivate this choice.

> I have added to the beginning of the introduction that coloc uses "only GWAS summary statistics", and added the following sentence to the end of the Introduction:
>
> "We use the summary statistics module of SuSiE, \texttt{susie_rss()}, so that the format of data currently expected by coloc, GWAS summary statistics for each trait and an LD matrix, is unchanged."

Figure 3. More help is needed in understanding Fig. 3, and why the results are presented in this way. If Fig. 3 is showing posterior probabilities shouldn't the bar heights always add up to 1? What is the "mask_it" column? And more discussion is needed on the results, and what this tells us about the performance of the different methods. In particular, what do the results in Fig. 3 tell us about when susie is expected to be an improvement over the other methods?

> Fig 3 is showing average posterior probabilities, where the average is taken over all simulations run. This is explained in the legend in the sentence
>
> "The total height of each bar represents the proportion of comparisons that were run for that variant pair, out of the number of simulations run, and typically does not reach 1 because there is not always power to perform all possible tests."
>
> In the case of coloc-single, each dataset will have exactly one comparison performed, and thus total bar heights over all 5 columns (AA,BB,AB,BA,?) will sum to 1. In coloc-cond one or more than one comparison will be performed per dataset, and therefore bar heights will sum to at least 1. In coloc-susie, a comparison is only made if credible sets are detected in both simulated datasets, and when this is not the case, that simulation contributes 0 to any posterior probability but 1 to the denominator. On the other hand, in some datasets, more than one credible set is identified, thus total bar heights could be less than or greater than 1, depending on data structure.
>
> Mask_it was not defined, as I had intended to drop masking from this paper. It performs comparably to conditioning, as originally reported and figure 3 (now figure 1) is already complicated enough, so I have instead removed the mask columns from the figure.
>
> First paragraph of Results describing this figure has been revised to be more explicit about the scenarios where SuSiE-based coloc outperforms all other options
>
> "When when both traits really did contain only a single causal variant, we found that single coloc generally performed best (top two rows of Fig 1). SuSiE-based analysis appeared to lose a little power (lower bar heights indicating fewer comparisons performed) but was equally accurate amongst comparisons performed. The situations when coloc-SuSiE did not perform any comparisons corresponded to cases where SuSiE did not identify any credible sets for one or both traits (Fig 2). A hybrid approach, running coloc-SuSiE if possible, and coloc-single if not outperformed any other strategy. When either one or both traits had two causal variants (bottom two rows of Fig \ref{fig:simstrat}), SuSiE outperformed all other methods in terms of accurately calling ``AB'' comparisons distinct (H_3) rather than shared (H_4) and performed as many or more comparisons than the other coloc methods. Hybrid SuSiE-single-coloc was very similar to SuSiE-coloc, or marginally better."
>
> I added two new figures to help communicate more clearly that susie is likely to lead to an improvement. First, Fig 2 shows that when coloc-susie does not perform any comparisons it is when GWAS signals are less strong, and suggest using coloc-single in this case. New Fig 4 looks at the relative ability of the coloc approaches described to improve upon single trait fine mapping, according to the criterion of whether the posterior probability of causality at the causal variant increases, and here coloc-SuSiE is shown to clearly outperform conditioning approaches when H4 (colocalisation) is inferred with probability >0.9.

Figure 4. Figure 4 is an interesting example. Where does it come from? We also need more details about the example (number of SNPs, chromosome, *etc*). When you show the logBF, is this base e or base 10? If you use log10 for p-values you should do the same for the BFs. When you say “SuSiE analysis of the same data finds one signal in trait 1”, are you using “signal” to mean a credible set (CS)?

Yes, I am using signal to mean the variant tagged by a credible set, but agree this isn’t clear, and have edited this legend to say credible set instead of signal. The BF was indeed given to log base e and is now shown as log base 10 and clarified to be so in the caption. This is an example hand picked from simulations of 1000 SNPs to illustrate a specific issue and happens to come from a region on chromosome 18. I agree the number of SNPs is useful information, and have added this together with the information that MAF>0.01 across them to the legend. I don’t think knowing this simulated example is from chromosome 18 is useful for a reader, but have added the full data behind this plot and supplementary figure 3 as a new supplementary data item, so the LD structure, SNP identities etc can be explored.

All the results are on simulated data. Motivated by your simulations, please present an example where using coloc + susie would improve colocalization of a real GWAS hit for two complex traits.

My difficulty with meeting this request is that in real data examples where coloc + susie finds a different result to coloc+conditioning, I would not be able to definitively declare which was an improvement on the other. Therefore I cannot provide such an example. The point of the simulations was to examine whether coloc+susie was an improvement *on average* when the ground truth was known, and I believe this was found to be the case where two causal variants exist for at least one trait.

Other comments:

Before “Adaptation of coloc approach” please briefly summarize coloc. What are the inputs and outputs? What are the summary statistics needed to run coloc? Also, please summarize what susie_rss does since it is not published.

I have revised the introduction to make clear that coloc requires only simple summary statistics

“.. calculated from GWAS effect estimates at each SNP and their standard errors[2].” (paragraph 1)

that this was extended by coloc conditioning approaches to require LD also

“we allowed for multiple causal variants in coloc by using conditional regression to distinguish lead variants, with the added requirement of supplying an LD matrix for the variants under test.“ (paragraph 3)

and that this was the motivation for susie_rss because it can work with the same information

“We use the summary statistic module of SuSiE, susie rss(), so that the format of data currently expected by coloc, GWAS summary statistics for each trait and an LD matrix, is unchanged.”

I summarise susie_rss only briefly, as it is still under development and I am cautious not to say anything which may be inaccurate in the near future

“While SuSiE is written in terms of the full genotype matrix, it has been extended to require only summary statistics by combination with a “regression with summary statistics” likelihood formulation [12].”

Are there other colocalization methods that might help with the problems investigated here? (But perhaps have additional limitations such as having high computational cost.) Please address this in the abstract and introduction.

> I now introduce alternative colocalisation methods in the Introduction. Different methods define colocalisation differently, so, no, I don't think there are other colocalisation methods that directly address this problem. eCAVIAR is the closest, but doesn't allow using prior expectations that colocalisation may be more likely than neighbouring independent causal variants.
>
> "Alternative methods for colocalisation have been developed which do not make this assumption. eCAVIAR[4] uses the CAVIAR[5] approach (which accommodates multiple causal variants) to fine map each trait, and gives probabilities that any variant is causal for both traits as the product of the single trait causal probabilities. However, this treats causality at each trait as independent events, when there is abundant evidence that a SNP causal for one trait is more likely to be causal for another. Alternatively, HEIDI/SMR [6] uses a frequentist framework, treating the null hypothesis as colocalisation, and rejecting this when there is evidence against. Here, multiple causal variants are dealt with by requiring colocalisation across all causal variants in a region, and that the ratio of effects of each causal variant on the two traits is constant across variants. Unlike these, coloc works with a single pair of causal variants at a time, and explicitly allows incorporating any expectation that causal variant are likely to be shared through prior probabilities."

To aid in reviewing the results, please give more memorable names to the hypotheses; it is hard to remember what H_1, H_2, … mean. Maybe H_none, H_{only 1}, H_{only 2}, H_diff, H_same?

> These names for the hypotheses have been used consistently since first proposed in 2014 and in subsequent applied papers that have cited coloc. I am reluctant to introduce new terminology now. Instead, I have restated the definitions in the text and figure legends where appropriate. For example, in the legend for figure 3:
>
> "Recall that H_0 indicates no associated variants for either trait, H_1 and H_2 a single causal variant for traits 1 and 2 respectively, H_3 and H_4 that both traits are associated with either distinct or shared causal variants, respectively."

For "Analysis compared different approaches…" in "Simulation strategy" please connect the methods to the labels used in Fig. 3.

> Each line now labelled using the same terms used in fig 3.

The figures should be uploaded at a higher resolution, or using a vector graphics format. Currently they are a bit difficult to view.

> The figures were saved at resolution of 300 DPI, and appear clear here, but PLOS does this horrible thing of showing you a low res version unless you click "download" on the figure.

I was able to install the "susie" variant of the coloc R package. Could you please provide a example or vignette illustrating how coloc.susie() works? (Perhaps drawn from the example presented in Fig. 4?)

> The package contains a vignette, also available at https://chr1swallace.github.io/coloc/articles/a06_SuSiE.html

"We used lddetect[9] to divide the genome into approximately LD-independent blocks, and extracted haplotypes from the EUR samples in 1000 Genomes phase 3 data, consisting of 1000 contiguous SNPs with MAF > 0.01." Should this be genotypes (not haplotypes)?

> This is now clarified:
>
> "We downloaded haplotypes for EUR samples in the 1000 Genomes phase 3 data\cite{1000_Genomes_Project_Consortium2015-lb}, phased by

IMPUTE2\cite{howie_impute2}, from \url{https://mathgen.stats.ox.ac.uk/impute/1000GP_Phase3.html}."

Please cite the 1000 Genomes paper when mentioning use of the 1kg data.

Now cited as above.

Reviewer #2: Review of Wallace (revised)

Thank you for the responsiveness to issues raised in the previous review.

I have just two points to be addressed.

1. As noted, Susie is under active development, and since v0.10.1 (March 16th 2021) the susie_rss function no longer performs eigen-decomposition of R. This fact could be noted in the discussion, and the version of susie used to produce the results reported here should be reported.

The new version I used appears considerably faster (v 0.11.42) - thank you for the continued development. I have rerun all analyses, and removed discussion of trimming and eigen decomposition because I don't think it is needed.

With regards versions, I have added to the end of the Methods:

"Results in this manuscript were generated using R version 4.0.4 with packages susieR version 0.11.42 and coloc version 5.1.0."

2. I found Figure 1 hard to read. Most of the ink is not very informative, and one has to read the actual numbers to extract the information. Also change in total PIP is probably less relevant than changes in individual PIPs (eg if all PIPs increase a very small amount, the total change can be big, but it probably doesn't matter much.) I think there should be better ways to convey the information. Possibly a scatterplot of PIPs for each SNP, with vs without trimming, might work - most of the points will presumably be near (0,0) but any outliers should be immediately apparent?

Figure 1 is now removed.

Have all data underlying the figures and results presented in the manuscript been provided?

Large-scale datasets should be made available via a public repository as described in the *PLOS Genetics* data availability policy, and numerical data that underlies graphs or summary statistics should be provided in spreadsheet form as supporting information.

Reviewer #1: Yes

Reviewer #2: None

PLOS authors have the option to publish the peer review history of their article (what does this mean?). If published, this will include your full peer review and any attached files.

If you choose "no", your identity will remain anonymous but your review may still be made public.

Do you want your identity to be public for this peer review? For information about this choice, including consent withdrawal, please see our Privacy Policy.

Reviewer #1: No

Reviewer #2: **Yes:** Matthew Stephens

In compliance with data protection regulations, you may request that we remove your personal registration details at any time. (Remove my information/details). Please contact the publication office if you have any questions.
