# Supplementary material for: A more accurate method for colocalisation analysis allowing for multiple causal variants
Source: PLoS Genet. 2021 Sep 29;17(9):e1009440. doi: 10.1371/journal.pgen.1009440 (PMC8504726; doi:10.1371/journal.pgen.1009440)
Supplement: Attachment — Submitted filename: coloc-susie revised, revised, response.pdf [file pgen.1009440.s009.pdf]

Reviewer #1: The manuscript is greatly improved since I last saw it. I have a few minor comments below, mostly to clarify some points that were not clear, but I trust the author will address them so I don't need to see another revision of the manuscript before publication.

Minor comments:

I didn't understand "ratio of effects" in this sentence: "Here, multiple causal variants are dealt with by requiring colocalisation across all causal variants in a region, and that the ratio of effects of each causal variant on the two traits is constant across variants."

> I have revised this sentence and added another to clarify:
>
> *Here, multiple causal variants are dealt with by requiring colocalisation across all causal variants in a region, and that the effects of each causal variant on the two traits is proportional. That is, if one causal variant has a two-fold greater effect on trait 1 compared to trait 2, then all other causal variants are assumed to also have a two-fold greater effect.*

I didn't follow this sentence, and how connected with the rest of the paragraph: "Thus, the user is presented with a list of tag SNPs per signal for each trait, and the matrix of pairwise posterior probabilities of H4 may be examined to infer which, if any, pairs of tags represent the same signal."

> Clarified as follows:
>
> *Thus, the user is presented with two lists of signals for each trait, and the $L_1\times L_2$ matrix of pairwise posterior probabilities of H_4 may be examined to infer which pair of tags, if any, represent the same signal.*

"This situation is confusing, because the same signal in trait 1 appears to colocalise with different signals in trait 1." Should this read "…signals in trait 2"?

> Yes, corrected.

I didn't see where Fig. 2 is referred to in the text.

> Now referenced in
>
> *The situations when coloc-SuSiE did not perform any comparisons corresponded to cases where SuSiE did not identify any credible sets for one or both traits, which were likely to be examples with higher minimum p values (Fig 2).*

Why is there no green line in Fig. 3a? And why are there no green points show in Fig. 3a, d?

> Because trait 1 only has one causal variant, A. Now clarified in the legend:
>
> *Trait 1 has one causal variant, A, and trait 2 has two, A and B.*

For completeness, I think Fig. 3 should also show trait 1, susie signal 2? (And similarly for Fig. S2.)

> SuSiE does not find a second signal for trait 1, as is already stated in the legend:

SuSiE analysis of the same data finds one credible set in trait 1

In the Fig. 3 caption you should also make clear what the truth is (I recognize that this is given in the text).

Now added

Trait 1 has one causal variant, A, and trait 2 has two, A and B.

"S2 Fig shows an example where the stepwise approach is less able to correctly identify the separate signals." By "stepwise approach" do you mean cond_abo?

Yes. Now clarified this paragraph to say explicitly which of cond_it and cond_abo I refer to each time.

The example in S2 Fig seems interesting and instructive—maybe it is worth putting in the main text? If I understand correctly, one difference is that susie iteratively improves the fit, whereas cond_abo does not iterate—it conditions B on A, then A on B, then stops. So perhaps one important improvement in susie is that it iteratively improves the fit until convergence? Perhaps this could explain why susie better identifies the signals in S2 Fig?

I think the difference is that susie fits a single joint model to the data, addressing the question "which single model (possibly with more than one causal variant) best explains the data. Whereas cond_abo is iterative, asking first "what is the strongest signal?" then, "what is the strongest signal after conditioning the first out". This is a procedure known to induce errors and to be less robust than fitting a single model.

I was conscious not to have too many display items, but would be happy to move this figure to the main text if the editor agrees.

"However, when no credible sets can be detected with confidence by SuSiE, single-coloc may still be able to make some inference." Do you know why susie fails in these cases? If there is an explanation, it would be helpful to add it here. I'm guessing that this failure occurs in cases where the support for association is not strong? On the surface the need for the "hybrid" method is a bit surprising, but there could very well be a good reason for it.

Yes, I believe Figure 2 shows that it is regions with higher minimum p values where susie doesn't detect any signals. This is due to its (appropriately) skeptical prior that any random SNP is causally associated. I have clarified this in referencing Fig 2:

The situations when coloc-SuSiE did not perform any comparisons corresponded to cases where SuSiE did not identify any credible sets for one or both traits, which were likely to be examples with higher minimum p values (Fig 2).

Under "Availability", you might want to mention the susie vignette in the coloc package, which seems particularly helpful for those interested in applying the new susie-based coloc methods.

I have added this.
