# Supplementary material for: A more accurate method for colocalisation analysis allowing for multiple causal variants
Source: PLoS Genet. 2021 Sep 29;17(9):e1009440. doi: 10.1371/journal.pgen.1009440 (PMC8504726; doi:10.1371/journal.pgen.1009440)
Supplement: Attachment — Submitted filename: review wallace plos genetics.docx [file pgen.1009440.s005.docx]

This is a promising methodological contribution to the colocalization problem in GWAS. The central idea is intruiging, and the key results support the idea, but the way the results are presented is confusing, and the ideas and results raise a few questions.

Here are my main comments:

My colleagues (who helped me with parts of this review) and I had trouble following Fig. 3. Our confusion may be in part due to several typos. In the second row should there be an arrow from A to 2 rather than B to 1? In the third row should there be an arrow from A to 2 and no arrow from B to 1? And the sentence toward the end of Results should instead read, “susie seems to resolve this issue, with AB-like comparisons clearly having strongest posterior support for H3” (not H4)? Some explanations are missing, such as the bars in the plots (AA, BB, AB, etc), and how the key statistical quantities computed in coloc (the Bayes factors and posterior odds) relate to the plots (in particular, the heights of the different coloured bars). Also, the total height of the bars seems important, perhaps relating to power in some way, which is alluded to in the caption, but isn’t clearly explained.

If we are interpreting the bars correctly, I was confused by the AB / BA signals. I think the key is when you assume one causal per region then AB / BA will inevitably capture some H4 because AB / BA are simply wrong models. In short, some of the bars may be more important than others to “get right”, and the way it is presented it may be hard for the reader (and reviewer!) to focus on the differences across methods that are important—by “important,” differences that would matter in practice when applying coloc to real data sets. For example, in the 4th scenario (4th row of Fig. 3), the results for “cond”, “mask” and “susie_0” are pretty much the same, except for the case of AB and BA. The question is whether these differences among the methods matter in this case? The level of detail in Fig. 3 may be useful for getting a better understand for the behaviour of the different approaches, but is there a way to summarize these results in an evocative way, say, using ROC or precision-recall curves?

A minor point is the results do not seem to differ much across different numbers of SNPs (1000, 2000, 3000) and I wonder if it would be better to show the results for only one setting, and move the other two settings to the supplement).

There is an early emphasis on addressing the issue that susie is too slow to be run on large regions. On the surface, it seems reasonable to “trim” SNPs based on some safe cutoff (e.g., based on p-values). However, some elaboration is needed to justify having to take this step; consider that the computational complexity of susie is linear in the number of samples (n), SNPs (p), and number of “single-effects” (L), so in principle susie should be able to handle large data sets, with available memory typically being the greatest limitation. (We have run susie on data sets with as many as 12,000 SNPs.) Also, for data sets with large n, there is a summary-statistics version of susie (susie_rss). I’m not arguing against the trimming per se, but to motivate these steps it would be helpful to explain in more detail the challenges faced in using susie, for example, whether the high computational expense was due to slow convergence (which could happen when SNPs are very strongly correlated), or due to something else. I wonder if this trimming is in fact avoidable.

A larger question is raised by relaxing the assumption of one causal variant, and it surfaces as one attempts to define the colocalization problem. Perhaps it can be addressed by more careful definitions. For example, you state in the introduction, “Colocalisation is a technique used for assessing whether two traits share a causal variant in a region of the genome, typically limited by LD.” But when allowing for the possibility of multiple causal variables, hypotheses H0–H4 no longer cover all possible events. For example, perhaps H4 should be defined as, *“both traits are associated and share at least one causal variant?”* In short, based on the description you have given, there appears to be a mismatch between the coloc inferences and the susie inferences, which is to compute a posterior distribution over all 2^p combinations of causal variables (with the constrained that at most L SNPs can be included). That is—if I am not misunderstanding—coloc makes inferences at the SNP level, and susie makes inferences at the region level. I don’t see this necessarily as a fundamental issue, but more an issue of being more precise in definitions (and perhaps reminding us—the readers and reviewers—what coloc is actually doing).

Finally, the simulation setup lacks detail, e.g., Which 1kg data set was used? How many 1000 Genomes samples were used, and from which geographic regions? How were the effects and traits simulated? How were the regions chosen (e.g., are they regions near gene coding regions)? What are the MAFs of the SNPs?
