# Supplementary material for: A more accurate method for colocalisation analysis allowing for multiple causal variants
Source: PLoS Genet. 2021 Sep 29;17(9):e1009440. doi: 10.1371/journal.pgen.1009440 (PMC8504726; doi:10.1371/journal.pgen.1009440)
Supplement: Attachment — Submitted filename: plos genetics review.docx [file pgen.1009440.s007.docx]

I apologize for the delay in sending my feedback. The revised manuscript is improved and makes several points more clear, although there remain some gaps that need to be addressed. Please see below for my detailed comments. My intent is for my comments to be constructive and I hope they inspire further improvements.

**Parts of the introduction remain imprecise.** For example, you say, “This simple summation is enabled by the single causal variant assumption, which implies that each pair of variants being causal for the two traits are mutually exclusive events.” I’m not sure what you mean by this? I think the main message is that the calculations of the BFs are much simpler under the single-variant assumption, which I agree with. Also, “The approach proceeds by enumerating all variant-level hypotheses — the possible pairs of causal variants (or none) for the two traits — and the relative support for each in terms of Bayes factors.” But don’t you allow (in susie) for more than 2 causal variants? Maybe introducing some simple mathematical expressions to define what is meant by the “single-variant assumption” and how the BFs are computed with and without the single-variant assumption would be helpful to make this more precise.

**Computational effort of fine-mapping with summary statistics.** Quite a bit of the discussion in this manuscript is centered on susie being slow for large numbers of SNPs, and how to alleviate this issue. While not wrong, this discussion may be misleading. This issue is specific to using susie with summary statistics; one of the inputs to susie_rss will be a p x p matrix, where p is the number of SNPs. But, fundamentally, any regression-based fine-mapping method that takes as input a p x p matrix will have complexity at least O(p^2), making this an issue for any method that uses summary statistics (of course, as you point out, because susie_rss computes an eigenvalue decomposition of the p x p matrix, the complexity is potentially greater than O(p^2), and there may be room to improve this). By contrast, with individual-level data susie’s complexity is linear in n and p, which will be better than the summary data case when n < p. Related to this point, in the introduction please clarify that you are focussing on fine-mapping using summary data, and motivate this choice.

**Figure 3.** More help is needed in understanding Fig. 3, and why the results are presented in this way. If Fig. 3 is showing posterior probabilities shouldn’t the bar heights always add up to 1? What is the “mask_it” column? And more discussion is needed on the results, and what this tells us about the performance of the different methods. In particular, what do the results in Fig. 3 tell us about when susie is expected to be an improvement over the other methods?

**Figure 4.** Figure 4 is an interesting example. Where does it come from? We also need more details about the example (number of SNPs, chromosome, *etc*). When you show the logBF, is this base e or base 10? If you use log10 for p-values you should do the same for the BFs. When you say “SuSiE analysis of the same data finds one signal in trait 1”, are you using “signal” to mean a credible set (CS)?

All the results are on simulated data. Motivated by your simulations, please present an example where using coloc + susie would improve colocalization of a real GWAS hit for two complex traits.

Other comments:

Before “Adaptation of coloc approach” please briefly summarize coloc. What are the inputs and outputs? What are the summary statistics needed to run coloc? Also, please summarize what susie_rss does since it is not published.

Are there other colocalization methods that might help with the problems investigated here? (But perhaps have additional limitations such as having high computational cost.) Please address this in the abstract and introduction.

To aid in reviewing the results, please give more memorable names to the hypotheses; it is hard to remember what H_1, H_2, … mean. Maybe H_none, H_{only 1}, H_{only 2}, H_diff, H_same?

For “Analysis compared different approaches…” in “Simulation strategy” please connect the methods to the labels used in Fig. 3.

The figures should be uploaded at a higher resolution, or using a vector graphics format. Currently they are a bit difficult to view.

I was able to install the “susie” variant of the coloc R package. Could you please provide a example or vignette illustrating how coloc.susie() works? (Perhaps drawn from the example presented in Fig. 4?)

“We used lddetect[9] to divide the genome into approximately LD-independent blocks, and extracted haplotypes from the EUR samples in 1000 Genomes phase 3 data, consisting of 1000 contiguous SNPs with MAF > 0.01.” Should this be genotypes (not haplotypes)?

Please cite the 1000 Genomes paper when mentioning use of the 1kg data.
